# Scalp electrode placement accuracy for the canine electroencephalography array

**DOI:** 10.3389/fvets.2025.1543836

**Published:** 2025-06-20

**Authors:** Stephen Everest, Myles St-Denis, Robert Dony, Luis Gaitero, Alexander Zur Linden, Miguel A. Cortez, Thomas Parmentier, Fiona M. K. James

**Affiliations:** ^1^Department of Clinical Studies, Ontario Veterinary College, University of Guelph, Guelph, ON, Canada; ^2^ACCESS Specialty Animal Hospital, Royal Palm Beach, FL, United States; ^3^School of Engineering, College of Engineering and Physical Sciences, University of Guelph, Guelph, ON, Canada; ^4^Division of Neurology, Department of Paediatrics, Temerty Faculty of Medicine, University of Toronto, Neurosciences & Mental Health Program, Peter Gilgan Center Research Learning, SickKids Research Institute, Toronto, ON, Canada; ^5^Département de Sciences Cliniques, Faculté de Médecine Vétérinaire, Université de Montréal, Montréal, QC, Canada

**Keywords:** dogs, electroencephalography, epilepsy, seizures, standardized electrode placement, 10–20 system

## Abstract

**Introduction:**

Despite the common occurrence of idiopathic epilepsy amongst neurological conditions in dogs, electroencephalography (EEG), the gold standard for seizure detection, is relatively neglected. The use of EEG in veterinary medicine is rudimentary compared to that in human medicine, particularly with respect to the quantification of EEG electrode placement error, i.e., the accuracy of electrode placement relative to the diverse canine cortical topography.

**Methods:**

Therefore, we quantified the intra-observer EEG electrode placement error using a single canine EEG electrode placement array, on virtual models of head and brain created from archived computed tomographic scans of Brachycephalic (*n* = 5), Mesocephalic (*n* = 15) and Dolichocephalic (*n* = 5) dogs from breeds with archetypal skull conformation. For the Mesocephalic cohort, a stereotactic brain atlas was incorporated into the brain models to quantify electrode placement error via a universal coordinate system. As this was not possible for the Brachycephalic and Dolichocephalic cohort, instead electrode placement was described in relation to cortical landmarks.

**Results:**

Gaps in cortical coverage between cohorts were identified, such as poor coverage of the olfactory and frontal regions in the brachycephalic cohort and the parietal region in the Mesocephalic and Dolichocephalic cohorts. Quantitative analysis of electrode placement in the Mesocephalic cohort showed the minimum variance of electrode localization for the x coordinate of the F8 electrode (0.8 mm) and the greatest variance for the y coordinate of the Cz electrode (35.2 mm).

**Discussion:**

This is the first study to highlight the knowledge gaps regarding the accuracy of canine EEG electrode localization, differences in the array coverage across the diverse canine skull conformations, and the urgent need for a stereotactic brain atlas for specific canine skull conformations.

## 1 Introduction

Idiopathic epilepsy is one of the most common neurological conditions in dogs, with epilepsy affecting between 0.6–0.75% of the general population (1, 2). Electroencephalography (EEG), the only patient-side test that confirms seizure activity through its recording of cortical electrical activity is under-utilized in veterinary patients (3). This is contrary to human medicine where EEG is the readily available tool for the detection of seizures and diagnosis of epilepsy types (3). Furthermore, specific EEG findings are integral to human epilepsy syndromes, defined by the International League Against Epilepsy as “a characteristic cluster of clinical and electroencephalographic features, often supported by specific etiological findings (structural, genetic, metabolic, immune, and infectious)” (4).

Instead, in veterinary medicine, EEG has been the last recommended diagnostic test in the three tiers of confidence for diagnosing idiopathic epilepsy. Tiers I and II combine signalment and history with a battery of tests, including examinations, labwork, a magnetic resonance imaging (MRI) scan, and cerebrospinal fluid (CSF) analysis, altogether ruling out structural epilepsy. For tier III confidence in the diagnosis, an EEG must have identified ictal or interictal epileptogenic patterns (5). Most veterinary patients diagnosed with idiopathic epilepsy remain at tier II confidence level as observed in a recent survey where less than half of veterinary neurologists reported performing EEGs (6). Even this is likely an overestimate given the 35% response rate, with the results influenced to some degree by selection bias, as people with an interest in EEG or participating in epilepsy research would be more likely to complete such a survey. This survey further highlighted a considerable diversity of approaches to placing EEGs in dogs, reporting at least nine EEG electrode arrays in use. This complex variability in technique and protocol likely discourage the use of EEG in veterinary patients, along with low confidence or incomplete understanding on how to use or place EEGs in veterinary patients (6). Regardless of the reasons for not performing EEGs, increased EEG usage would improve confidence in idiopathic epilepsy diagnoses, further our understanding of veterinary EEGs, as well as support the diagnosis of specific epilepsy syndromes, likely to occur in dogs. In addition, accurate source localization would pave the way for veterinary epilepsy surgery. Source localization requires that both the forward and inverse problems be addressed; the forward problem identifies the cortical source of the signal detected by the EEG electrodes and the inverse problem determines which electrode(s) would detect a signal from a given cortical source (7).

Amongst the range of EEG electrode arrays in use, one was developed using a series of anatomical studies across brachy-, dolicho- and mesocephalic dog heads (8). Electrodes were placed on 80 dog cadavers using external bony landmarks; dissections then determined electrode localization in relation to cortical landmarks (8). The next steps for this array will be to account for intra- and inter-placer variability and confirm the repeatability of cortical coverage across the range of dog skull morphologies (9). While the accuracy of electrode placement was improved using MRI or computed tomography (CT)-based neuronavigation, this is clinically impractical and expensive, and variance estimates on localization error have yet to be robustly calculated (9, 10). There are suggestions of incomplete coverage of the frontal lobe, as well as systematic errors in the positioning of the other electrodes with manual placement (9, 10). An update to this array was proposed that used more frontolateral electrodes to improve frontal lobe coverage while performing unsedated recordings (11). To date, validation of this updated modified EEG array has not been performed. Specifically, localization error, the variation in EEG electrode placement in relation to cortical landmarks, has not yet been quantified in veterinary patients.

The aim of this study was the craniocerebral topographic examination of the modified EEG array as a first step towards solving the inverse problem in canine EEG (11). The objectives were: (1) to describe the location of the array’s electrodes with respect to cortical landmarks in cohorts of brachycephalic, mesocephalic and dolichocephalic dogs, (2) to quantify the EEG electrode localization error in a cohort of dogs while limiting inter-placer variation, and (3) to propose improvements to the array if needed (11). It was hypothesized that the modified EEG array would have well-distributed cortical coverage for all three dog head morphologies with minimal localization error for mesocephalic dogs.

## 2 Materials and methods

This retrospective observational descriptive cohort study was performed in accordance with guidelines of the Council on Animal Care and was approved by Animal Care Committee of the University of Guelph (Animal Utilization Protocol #4320). The institution’s digital picture archiving and communication system (PACS) was searched for magnetic resonance imaging (MRI) or computed tomography (CT) studies of the canine head obtained between February 2013 and March 2021, which were then cross-referenced with the electronic medical record.

### 2.1 Animals

The dogs’ brains and cranial cavities had to be grossly unremarkable on MRI or CT, and a CT study of the brain acquired with the bone and soft tissue algorithms had to be available. Medical records had to include the dog’s breed, age, and sex. Included dogs had to be over a year of age to ensure that growth plates had closed.

To reduce controversy regarding head conformation, dogs were included into each conformation cohort where the breed was commonly considered to fit. For the brachycephalic group, pugs, French bulldogs, English bulldogs and Boston terriers were considered acceptable. For the mesocephalic group, golden retrievers and Labrador retrievers were considered acceptable. For the dolichocephalic group, greyhounds and Italian greyhounds were considered acceptable. For the initial cohort for Objective 1, convenience samples of five brachycephalic dogs, five mesocephalic dogs, and five dolichocephalic dogs were selected after review of their medical records. For the expanded cohort for Objective 2, an additional 10 mesocephalic dogs were selected after similar medical record review. Dogs were excluded if recorded as mixed breed, or if their presenting complaint or aetiology altered normal brain or skull gross anatomy.

#### 2.1.1 Study protocol

**Figure 1:**
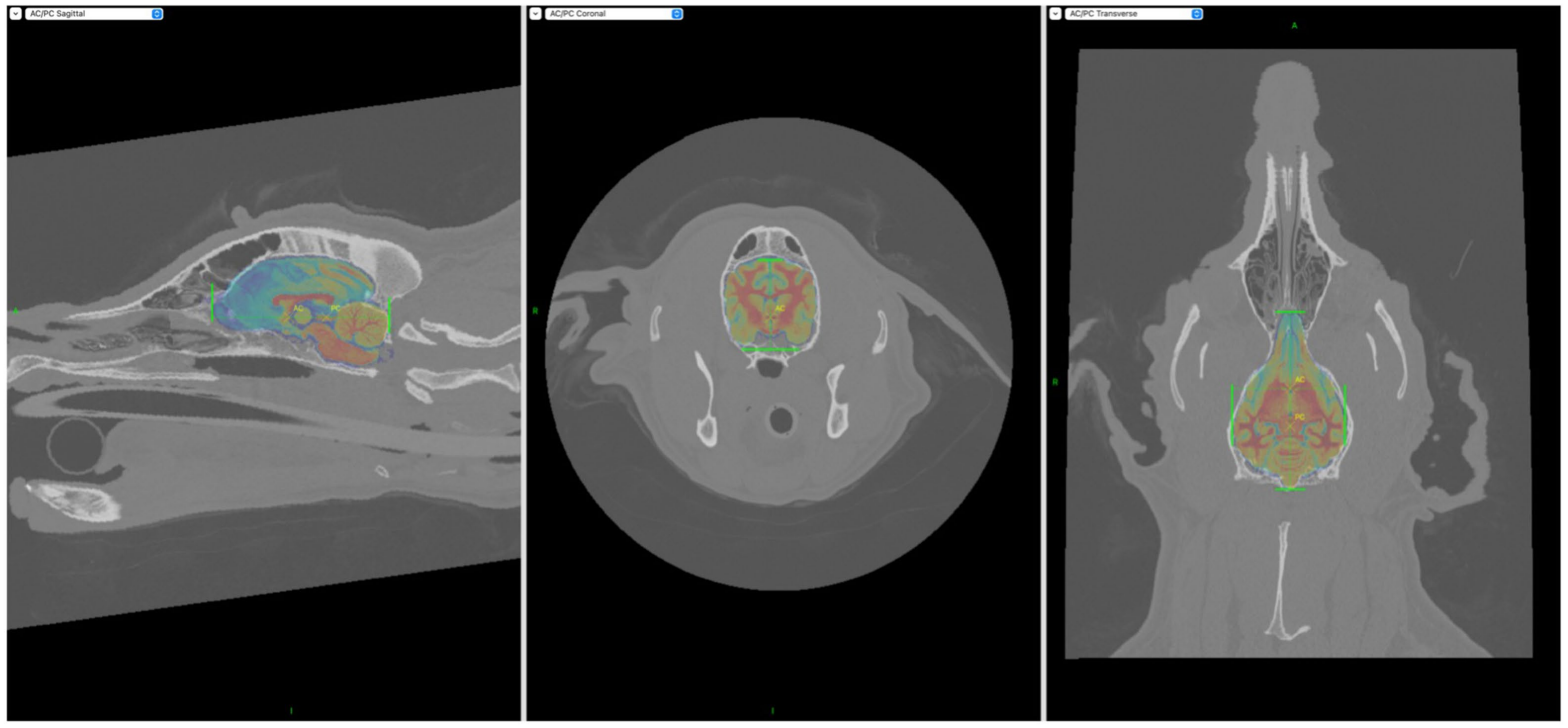
Screen captures demonstrating incorporation of the Johnson et al. (12) stereotactic brain atlas into the BrainSight CT models. Areas of interest had to be manually highlighted with the aid of the green markers seen above.

Each subject had three-dimensional (3-D) brain and skin models created using BrainSight [Rogue Research Inc., 2022; Version2.5b6 (70523)], an image-guided neuronavigation system appositely designed for veterinary use. After anonymization, the CT study bone algorithm images were imported into BrainSight. A previously published brain atlas (12) was then manually registered to the image set to apply a coordinate system common to all dogs (Figure 1). This common reference frame coordinate system enabled the quantitative assessment of electrode localization error in Objective 2. In this coordinate system, the x axis is the transverse plane, the y axis is the sagittal plane, and the z axis is the dorsal plane. Positive x is right, positive y is rostral, and positive z is dorsal. After it was confirmed that the brain atlas could only be applied to mesocephalic 3-D models, quantitative assessment of electrode localization error (Objective 2) was performed only on the expanded mesocephalic cohort of 15 dogs. The brachycephalic or dolichocephalic cohorts therefore only underwent qualitative assessment (Objective 1).

**Figure 2:**
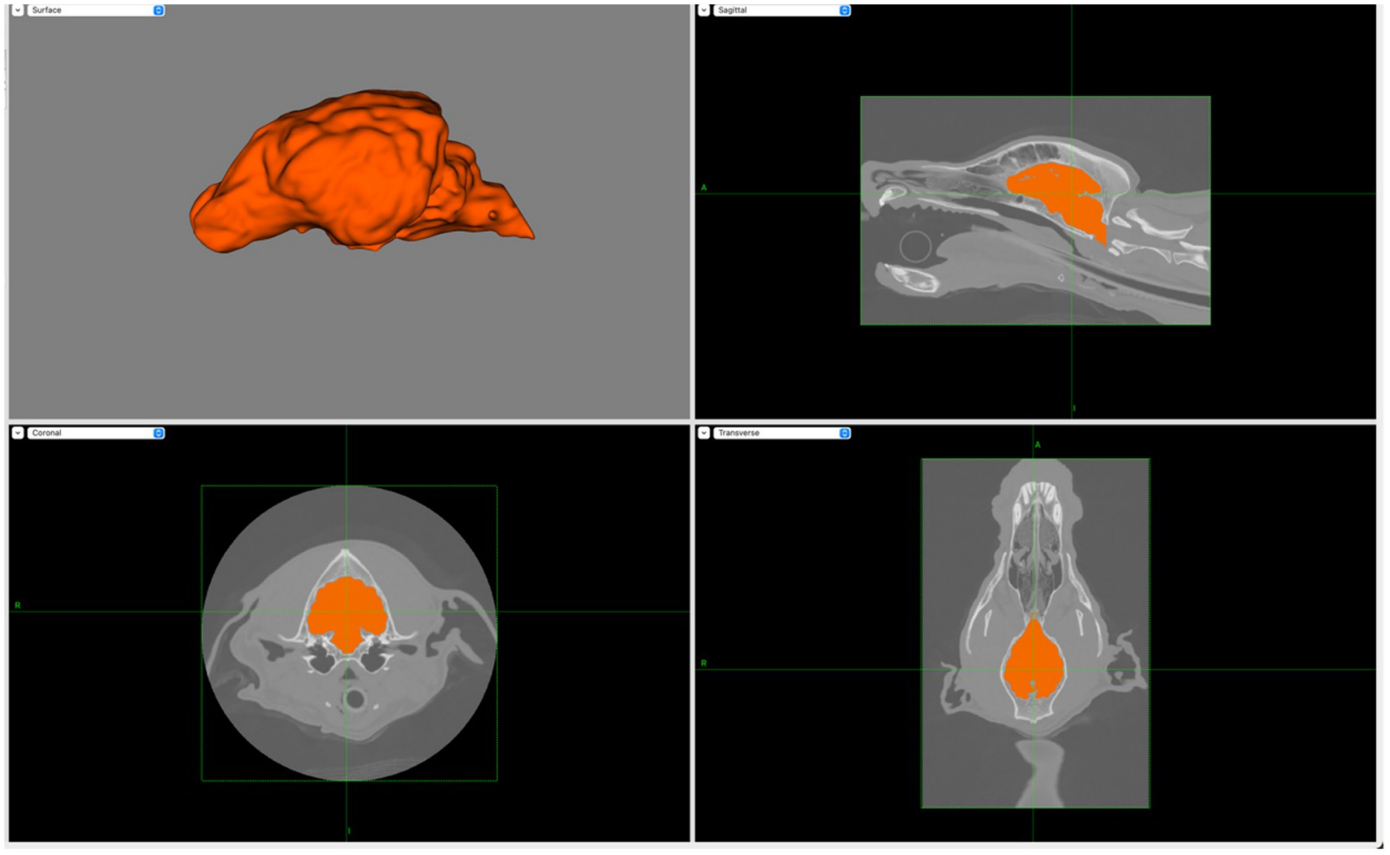
Screen capture from BrainSight demonstrating the creation of the brain layer (upper left) with the manual selection of brain ROIs using transverse (lower left), sagittal (upper right) and dorsal (lower right) planes. Notably, main sulci and gyri are visible on the brain layer.

Appropriate regions of interest (ROIs) were then manually selected (using the drawing and fill tools) to create two layers per dog- skin and brain layers. Figure 2 illustrates how the manual selection of the ROIs ensured accuracy of the layers.

Virtual markers for all electrodes were placed on the skin layer of each 3D model within BrainSight following the instructions for the modified electrode array by a single observer as shown in Table 1 (8, 11). These electrodes were F3/Fz/F4, F7/F8, C3/Cz/C4, T3/T4, O1/O2, and Pz, totalling 13 electrodes per dog. BrainSight trajectories were then used to determine the closest point on the brain layer for each skin layer electrode.

In the Brachycephalic and Dolichocephalic cohorts, the proposed brain atlas was not incorporated, and location of each electrode placement was described qualitatively by a neurology resident (SE) for Objective 1 with respect to the closest prominent brain feature, e.g., a named sulcus or gyrus according to de Lahunta et al. (13). For the expanded Mesocephalic cohort for Objective 2, the coordinates for each electrode were recorded (see Supplementary material).

**Table 1:** Instructions for placement of the EEG electrodes for the James et al. (11) array.

Electrode	Anatomic landmark
Ref	Midline, between medial canthi
Grd	Dorsal midline neck, 2–5 cm caudal to occipital protruberance
F7/F8	Zygomatic arch just caudal to the lateral canthus of both eyes
F3/F4/Fz	On the temporal lines caudal to the medial canthi and at midline
C3/C4/Cz	Halfway between F and O/P electrodes, in line with T electrodes
01/02/Pz	Transverse line between mastoid processes in line with F electrodes
T3/T4	Zygomatic arch, just rostral to the pinnal edge

### 2.2 Statistical analysis

**Figure 3:**
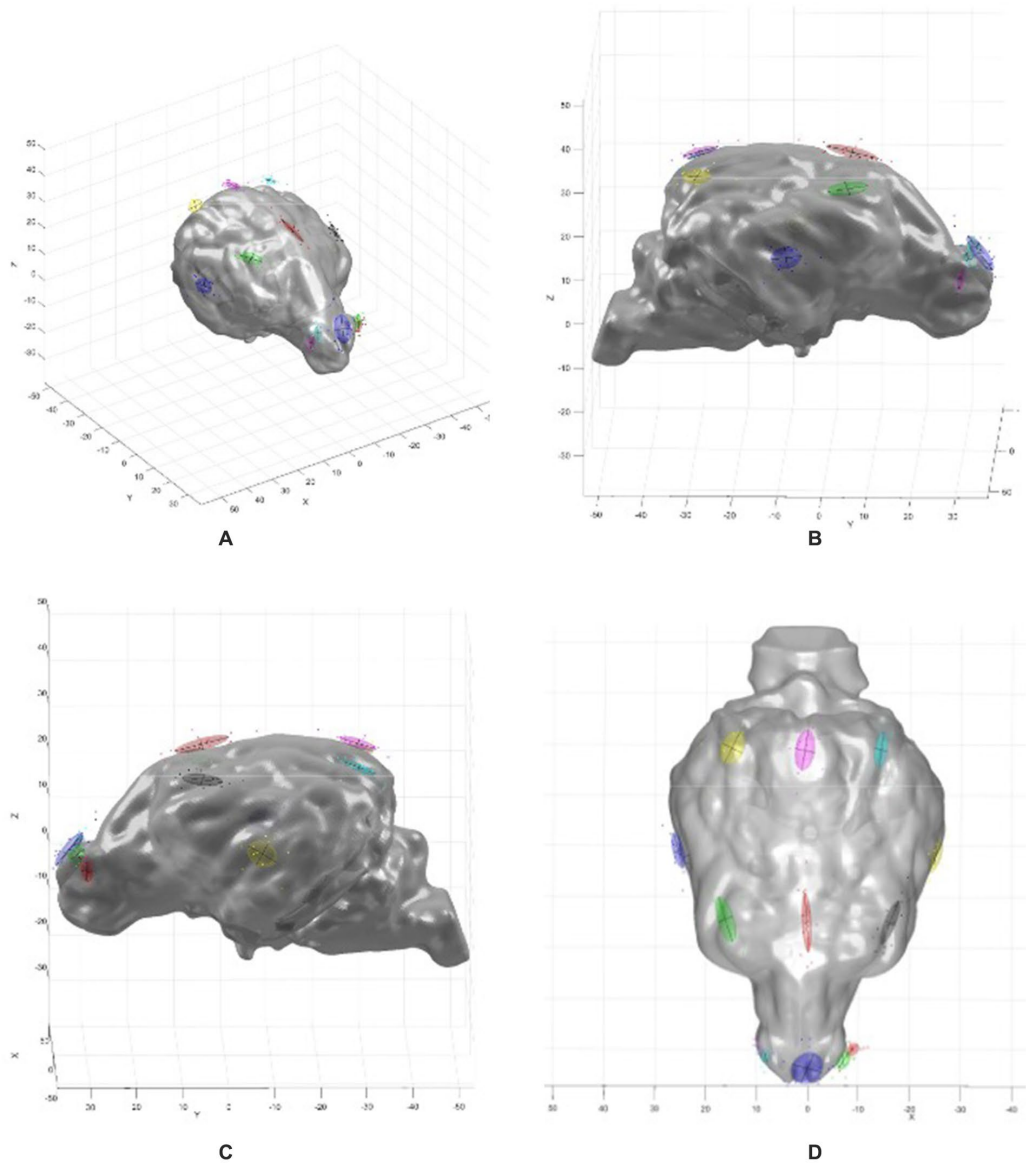
MATLAB 3-D graphs showing the brain model with the PCA overlay. **(A)** Oblique view, **(B)** right sagittal view, **(C)** left sagittal view, **(D)** dorsal view. Coloured ovoids indicate electrode localization errors, with locations corresponding to the electrode array map in Figure 2. The coloured ovoids are the 1 standard deviation contours of the electrode localization errors. The lines indicate the directions of variance for the two largest components.

For the qualitative analysis for Objective 1, simple descriptions were used. For the quantitative analysis for Objective 2, the brain surface coordinates for each of the electrodes placed virtually on each mesocephalic dog model were imported into MATLAB (MathWorks Inc., 1984–2022; R2022a Update 1 (9.12.0.1927505)). Principal component analysis (PCA) was performed in MATLAB (code provided in Supplementary materials) to calculate the average coordinates for each electrode as well as the electrode placement error and direction. A 3-D brain model was then created from a randomly chosen dog from the Mesocephalic cohort using the trisurf (surface) function in MATLAB. The PCA results were then overlaid for a visual representation of the craniocerebral topographic map of the electrode array and the localization error in three dimensions of each electrode in relation to the brain (Figure 3).

## 3 Results

### 3.1 Animals

In total 25 dogs were included in this study. Table 2 presents the breed, age and sex for all three cohorts. The mean age of the brachycephalic cohort was 5.2 years (range: 2–10 years) with three female spayed (FS) and two male neutered (MN) subjects. The mean age of the mesocephalic cohort was 6.5 years (range: 2–14 years), with 6 FS, 7 MN and 2 male entire (ME). The mean age of the dolichocephalic cohort was 9.2 years (range: 7–14 years), with 2 FS and 3 MN dogs.

**Table 2:** The breeds, age and sex for the three cohorts included in this study.

Conformation	Breed	Age	Sex
Brachycephalic	Boston terrier	5 years	Male, neutered
Brachycephalic	English bulldog	5 years	Female, spayed
Brachycephalic	Pug	10 years	Female, spayed
Brachycephalic	French bulldog	4 years	Male, neutered
Brachycephalic	French bulldog	2 years	Female, spayed
Mesocephalic	Golden retriever	5 years	Male, neutered
Mesocephalic	Golden retriever	3 years	Female, spayed
Mesocephalic	Labrador retriever	8 years	Male, neutered
Mesocephalic	Labrador retriever	10 years	Male, neutered
Mesocephalic	Labrador retriever	8 years	Female, spayed
Mesocephalic	Labrador retriever	11 years	Female, spayed
Mesocephalic	Labrador retriever	6 years	Male, neutered
Mesocephalic	Labrador retriever	14 years	Female, spayed
Mesocephalic	Golden retriever	4 years	Male, entire
Mesocephalic	Labrador retriever	3 years	Male, neutered
Mesocephalic	Labrador retriever	4 years	Female, spayed
Mesocephalic	Labrador retriever	2 years	Female, spayed
Mesocephalic	Golden retriever	10 years	Male, neutered
Mesocephalic	Labrador retriever	2 years	Male, neutered
Mesocephalic	Golden retriever	7 years	Male, entire
Dolichocephalic	Rough coated collie	9 years	Female, spayed
Dolichocephalic	Rough coated collie	7 years	Female, spayed
Dolichocephalic	Shetland sheepdog	8 years	Male, neutered
Dolichocephalic	Rough coated collie	8 years	Male, neutered
Dolichocephalic	Italian greyhound	14 years	Male, neutered

### 3.2 Qualitative study (objective 1)

Table 3 shows the closest prominent brain feature manually identified for each electrode in each dog in the three cohorts.

**Table 3:** Summary of the locations of the electrodes in the modified array in relation to nearest cortical landmarks in brachycephalic, mesocephalic and dolichocephalic dogs compared to the brain projection area of cortex expected from the modified array (8, 11).

Electrode	Brachycephalic	Mesocephalic	Dolichocephalic	Expected brain projection areas
F7	Rostral portion of the left rostral supra-sylvian gyrus	Olfactory bulb	Olfactory bulb	Agranular cortex: precruciate gyrus
F3	Rostral portion of the left rostral supra-sylvian gyrus	Olfactory bulb	Olfactory bulb	Agranular cortex: precruciate gyrus
Fz	Rostral portion of the longitudinal fissure at the level of the frontal gyri	Olfactory bulb	Olfactory bulb always lateralised slightly to either side	Agranular cortex: precruciate gyrus
F4	Rostral portion of the right rostral supra-sylvian gyrus	Olfactory bulb	Olfactory bulb	Agranular cortex: precruciate gyrus
F8	Rostral portion of the right rostral supra-sylvian gyrus	Olfactory bulb	Olfactory bulb	Agranular cortex: precruciate gyrus
T3	Ventrocaudal aspect of the left ectosylvian gyrus	Curvature of the sylvian gyrus at the most caudal aspect of the pseudosylvian fissure on the left	Most rostral aspect of the left ectosylvian gyrus	Granular cortex (temporal area): pseudosylvian fissure
C3	Caudal aspect of the middle supra-sylvian gyrus lateralised to the left	Precruciate gyrus on the left	Left precruciate gyrus	Parietal cortex: rostral part of the ectomarginal gyrus [based on the synonymous P3 electrode described by Pellegrino and Sica (8)].
Cz	Longitudinal fissure just cranial to the rostral aspect of the endomarginal gyrus	Rostral longitudinal fissure, sometimes lateralised slightly to either the left or right at the level of the precruciate gyrus	Longitudinal fissure at the level of the precruciate gyrus	Parietal area: Brain longitudinal fissure
C4	Caudal aspect of the middle supra-sylvian gyrus lateralised to the right	Precruciate gyrus on the right	Right precruciate gyrus	Parietal cortex: rostral part of the ectomarginal gyrus [based on the synonymous P4 electrode described by Pellegrino and Sica (8)].
T4	Ventrocaudal aspect of the right ectosylvian gyrus	Curvature of the sylvian gyrus at the most caudal aspect of the pseudosylvian fissure on the right	Most rostral aspect of the right ectosylvian gyrus	Granular cortex (temporal area): pseudosylvian fissure
O1	Middle of the ectomarginal gyrus on the left	Caudal aspect of the left marginal gyrus	Middle of the left ectomarginal gyrus	Granular cortex (occipital area): marginal gyrus/occipital gyrus
Pz	Longitudinal fissure, slightly lateralised to either the left or right marginal gyrus.	Caudal aspect of the longitudinal fissure, sometimes mildly lateralised to either side	Caudal aspect of the longitudinal fissure, often lateralised mildly to either the left or right	Occipital area: Brain longitudinal fissure [based on the synonymous Oz electrode described by Pellegrino and Sica (8)].
O2	Middle of the ectomarginal gyrus on the right	Caudal aspect of the right marginal gyrus	Middle of the right ectomarginal gyrus	Granular cortex (occipital area): marginal gyrus/occipital gyrus

Brain projection areas were defined as the cortical landmarks in closest proximity to each electrode as previously utilized by Pellegrino and Sica (8) and Rogers et al. (10).

**Figure 4:**
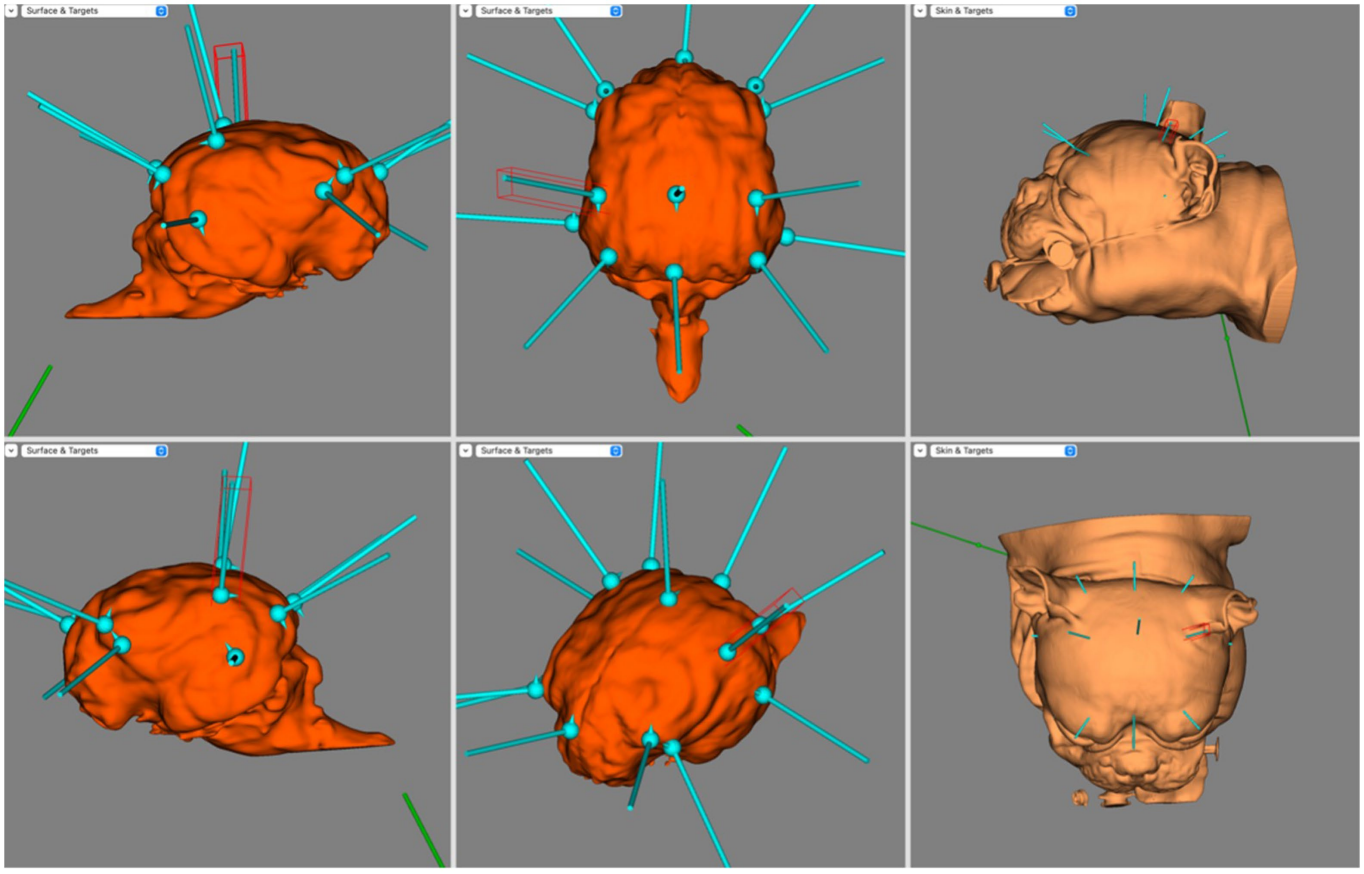
BrainSight screen capture demonstrating the electrode coverage in a brachycephalic dog model. Top left is the right sagittal view of the brain, bottom left is the left sagittal view of the brain, top centre is the dorsal view of the brain, bottom centre is the oblique view of the brain, top right is the left lateral view of the skin model and bottom right is the dorsal view of the skin model.

Figure 4 illustrates the electrode coverage in one of the brachycephalic subjects. In the Brachycephalic cohort, F7 and F3 were both over the rostral portion of the left rostral supra-sylvian gyrus, and F8 and F4 were over the rostral portion of the right rostral supra-sylvian gyrus. Fz generally landed over the rostral portion of the longitudinal fissure at the level of the frontal gyri. It was typically mildly lateralised to either the left or right frontal gyrus. The T3 and T4 electrodes were positioned over the ventrocaudal aspect of the left and right ectosylvian gyri, respectively. C3 and C4 covered the caudal aspect of the middle supra-sylvian gyrus lateralised to the left and right, respectively. Cz was over the longitudinal fissure just cranial to the rostral aspect of the endomarginal gyrus. The O1 and O2 electrodes had a tendency to localize in the middle of the ectomarginal gyri on the left and right, respectively. Finally, Pz usually landed over the longitudinal fissure, slightly lateralised to either the left or right marginal gyrus.

**Figure 5:**
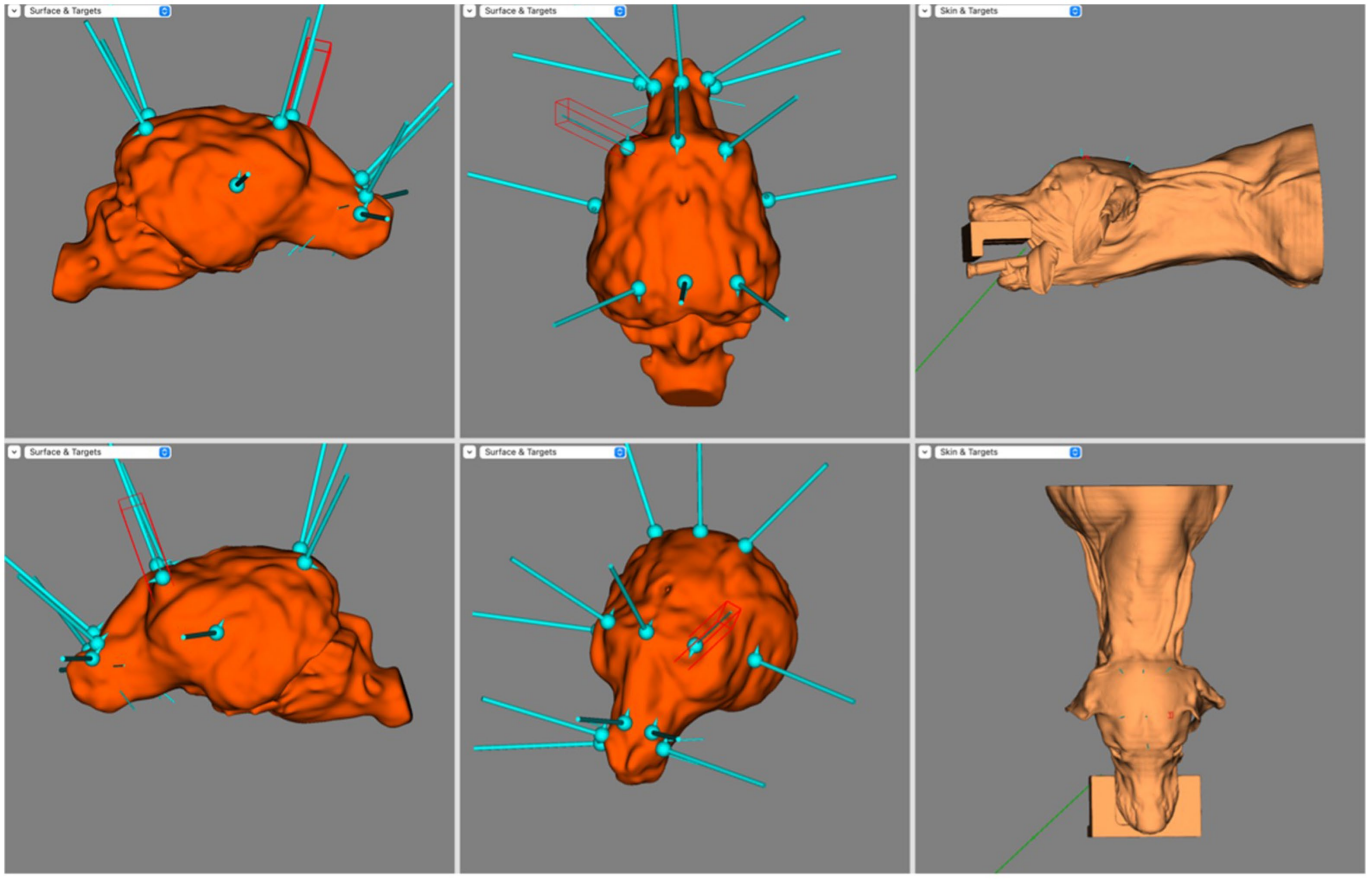
Screen capture from BrainSight demonstrating electrode coverage in one of the mesocephalic dog subjects. Top left is the right sagittal view of the brain, bottom left is the left sagittal view of the brain, top centre is the dorsal view of the brain, bottom centre is the oblique view of the brain, top right is the left lateral view of the skin model and bottom right is the dorsal view of the skin model. Electrode coverage in Dolichocephalic dogs is illustrated in Figure 6. All F electrodes in Dolichocephalic dogs were over the olfactory bulb. Fz was always lateralised slightly to either side. T3 and T4 covered the most rostral aspect of the left and right ectosylvian gyri, respectively. C3 and C4 were over the precruciate gyri brilaterally, and Cz was over the longitudinal fissure at the level of the precruciate gyrus. O1 and O2 were both in the middle of the ectomarginal gyrus and Pz was over the caudal aspect of the longitudinal fissure, often mildly lateralised to either the left or right.

Figure 5 illustrates the electrode coverage in Mesocephalic dogs, using a single subject from the cohort. When placing the F3, Fz, or F4 electrodes, it was noted that all these generally covered the olfactory bulb. In placing F7 and F8 it was noticed that the corresponding cortex was markedly different with even small skin surface location changes; a difference of 2–3 mm caudally meant these electrodes mapped to either the prorean or precruciate gyri of the frontal lobe instead of the olfactory bulb. The T3 and T4 electrodes landed at the curvature of the sylvian gyrus at the most caudal aspect of the pseudosylvian fissure bilaterally. The C3 and C4 electrodes were over the precruciate gyrus bilaterally, and the Cz electrode was over the rostral longitudinal fissure, sometimes lateralised slightly to either the left or right at the level of the precruciate gyrus. O1 and O2 covered the caudal aspect of the marginal gyrus bilaterally. Finally, Pz was over the caudal aspect of the longitudinal fissure, sometimes mildly lateralised to either side.

### 3.3 Quantitative study (objective 2)

The common reference coordinate system could not be applied accurately in all Brachycephalic and Dolichocephalic subjects. There was no transformation that adequately aligned the atlas brain with the subject scans. Therefore, only electrode placement in the Mesocephalic cohort was studied quantitatively after additional scans were included to expand the cohort to 15 subjects.

The x, y and z coordinates of the array electrodes for each subject are shared in Supplementary materials. The means and variances of the coordinates for each electrode are reported in the penultimate and bottom rows, respectively. The greatest variance was for the y coordinate of the Cz electrode (35.2 mm), whereas the smallest variance was for the x coordinate of the F8 electrode (0.8 mm). From greatest to least variance, the electrodes ranked: Cz, C4, C3, Pz, Fz, T4, O1, O2, T3, F7, F4, F3 and F8. The mean variance was 6.62 mm. It was noted whilst placing the virtual electrodes that F7 and F8 was particularly sensitive to location. If placed immediately adjacent to the lateral canthus of the eye the electrodes landed over the olfactory bulb, however if placed 2–3 mm caudally the electrodes landed over either the prorean or precruciate gyri, thus introducing considerable variation.

### 3.4 Objective 3

Based on the combined findings of Objectives 1 and 2, there was no coverage of the olfactory bulb in the Brachycephalic cohort, whereas in both the Mesocephalic and Dolichocephalic cohort all five F electrodes were over the olfactory bulb. It was also noted that in the Brachycephalic cohort the C electrodes were relatively more caudal than in the Mesocephalic and Dolichocephalic cohort. In the Brachycephalic cohort these electrodes were all in the parietal region, whereas in the Mesocephalic and Dolichocephalic cohort they were all in the frontal region at the level of the precruciate gyrus.

## 4 Discussion

This study is the first to measure values for the electrode localization error in a canine EEG electrode array. A recent study by Rogers et al. similarly used neuronavigation to provide likelihoods of any particular electrode overlapping the expected brain projection areas of the cortex, but did not measure the absolute localization errors (10). Notably, the arrays in the present study and the Rogers study were both based on the same original array and both found similar deviations from the target cortical area (8).

The study performed by Rogers et al. (10) found that 32% of the electrodes did not align with the proposed brain projection area, the majority of which were the Fp electrodes which tended to divert to the prorean and postcruciate gyri. This may be consistent with the challenges faced when placing the F7 and F8 electrodes in the present study, where only a minor movement of the electrode caudally resulted in the nearest cortical landmark being the prorean or precruciate gyrus rather than the olfactory bulb in the mesocephalic cohort. The Rogers study also reported that P electrodes could divert to the medial aspect of the ectomarginal gyrus, the caudal part of the ectomarginal gyrus and the precruciate gyrus of the frontal lobe, as well as to other cortical lobes. They noted the O electrodes diverted to the medial aspect of the ectomarginal gyrus of the parietal lobe, which was similar to the finding in the present study. The T electrodes could divert to the ectosylvian gyrus, ectomarginal gyrus, sylvian gyrus and composite gyrus, and some to the occipital gyrus (10). In the present study, these electrodes were over the ectomarginal gyrus in both Brachycephalic and Dolichocephalic cohorts, but tended to fall over the curvature of the sylvian gyrus in Mesocephalic dogs.

Pellegrino and Sica (8) proposed their EEG array in 2004, using the nomenclature of the 10–20 system used for people, on the basis of painstaking anatomical dissections. Instructions based on palpable bony landmarks were generated for the placement of 12 electrodes plus reference and ground. The present study and the recent Rogers et al. (10) study were not the first to assess the Pellegrino and Sica (8) array. An unpublished abstract presented by Daniel et al. (9) used both gross and virtual dissection to determine electrode location in a single cadaver. Whilst this confirmed good agreement between gross and virtual dissection, it found incomplete coverage of the frontal lobes and caudal displacement of the electrodes placed over the parietal lobe.

The improvement in accuracy of placement with MRI neuronavigation was supported by a later unpublished abstract presented by Poma et al. (14) The discrepancy of electrode positioning found by three different groups using combinations of gross dissection and neuronavigation indicates that there is considerable inter-observer electrode placement variability in electrode positioning (8, 9).

Whilst the use of MRI or CT-guided neuronavigation improves the accuracy of EEG electrode placement compared to using palpable bony landmarks, this approach requires access to advanced diagnostic equipment that is orders of magnitude more costly than the EEG procedure itself. Neuronavigation suites like BrainSight offer the main benefit of accurate virtual ‘dissections’. These virtual 3-D models facilitated both qualitative and quantitative assessment of EEG electrode placement in this study. The newly incorporated canine brain atlas in BrainSight allowed the coordinates of each electrode to be recorded in a common reference coordinate system (12). This meant that, for the first time, the variance of electrode localization errors could be calculated in x, y, and z axes in an EEG electrode array, which is an important step towards the development of epilepsy surgery for dogs.

This study determined that the variance ranged from the y axis of the Cz electrode (35.2 mm) to the x axis of the F8 electrode (0.8 mm). In humans the average localization error when using the 10–20 array has been determined to be 13–17 mm depending on the number of electrodes used (15). Other factors reported to affect electrode localization error in people include volume conduction of the head when using forward modelling, requiring more complex modelling than presented in this study (16). It has also been proposed that accuracy may be worse in older patients compared to young patients due to brain atrophy (17). In the present study, the Cz electrode may have had the largest localization error in the sagittal plane partly because the correct placement of the C electrodes is dependent on accurate placement of the F and O/P electrodes, as well as the T electrodes. This differs from the F7 and F8 electrodes which have arguably the easiest anatomic landmark to identify, being the lateral canthus of the eye over the zygomatic arch. It is difficult to determine whether these findings fully agree with the findings of Daniel et al. (9) or Rogers et al. (10) as both studies looked at the original Pellegrino and Sica (8) array, whereas this study did not use Fp electrodes. The Rogers et al. (10) study found that 92% of the electrodes projecting to variable cortical regions were the Fp electrodes, which are not included in the James et al. (11) array. It should also be noted that there is currently no consensus on electrode nomenclature in veterinary patients. For example, the Pellegrino and Sica (8) P3 and P4 electrodes are the same location as the James et al. (11) C3 and C4 electrodes, respectively. In the human 10–20 array the C3 and C4 electrodes are in line with the ears and the T3 and T4 electrodes. It is also noted that the C3 and C4 electrodes in the array proposed by James et al. (11) tended to cover the regions of the precruciate gyrus, similarly to the same electrodes in the human 10–20 array, which cover primarily the precentral and poscentral gyri. With this in mind the nomenclature utilized in the James et al. (11) array is more consistent with the nomenclature used for the human 10–20 system array.

Performing principal component analysis in this study enabled the direction of the localization error to be illustrated graphically in MATLAB (Figure 5). It is a limitation of this study that this localization error is a measure of the intra-observer variability, that is, all electrode positioning on the virtual models was performed by a single observer. That a single observer was placing the virtual electrodes meant this study did not address inter-observer variability, which would be required to fully establish the average localization error when placing this array in dogs. In addition, the virtual nature of this study means further investigation of electrode placement is required to see if the virtual results are reflected in live dogs. This is important taking into account that there are variables in live dogs that do not occur in virtual models. An example of this is skin elasticity, an essential variable to consider as it is something that is not consistent across dog breeds. That is, skin elasticity is likely to have more of an impact on electrode placement in breeds such as shar peis compared to breeds with relatively less integument. This may have a considerable impact on electrode localization error, particularly when taking into account movement of the dog that may occur when placing the electrodes or repositioning when the patient is under general anaesthesia for example. Finally, the placement of the F7 and F8 electrodes on the virtual model indicated that there could be significant variation in their projection to the cortex based on how rostrally or caudally they are positioned on the skin. This is of concern because it highlights a significant risk of intra-observer variability even when placing electrodes virtually. It is particularly important to bear this in mind if attempting to identify a seizure locus to facilitate epilepsy surgery.

**Figure 6:**
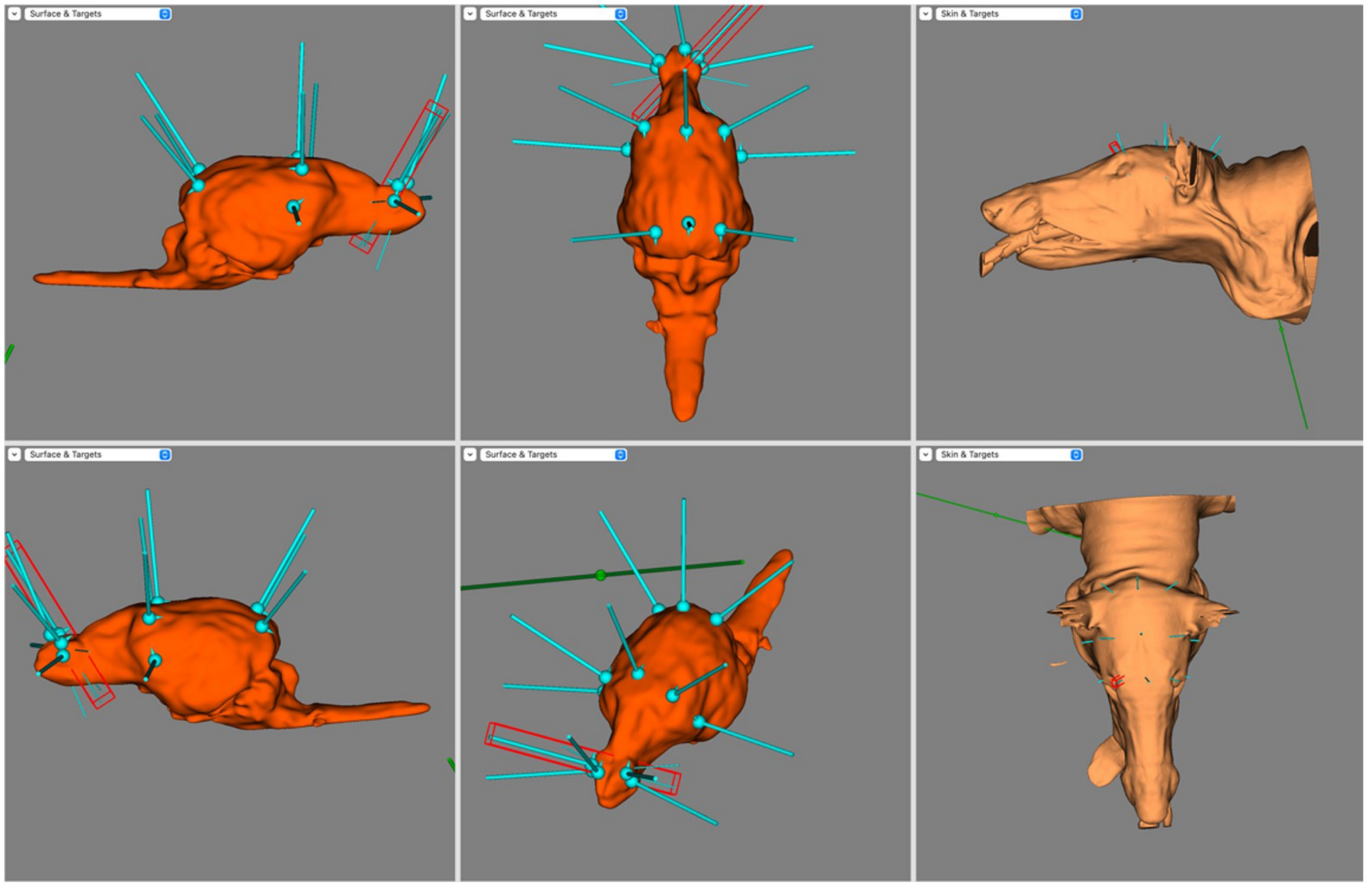
Screen capture from BrainSight demonstrating a typical electrode distribution in a subject from the Dolichocephalic dog cohort. Top left is the right sagittal view of the brain, bottom left is the left sagittal view of the brain, top centre is the dorsal view of the brain, bottom centre is the oblique view of the brain, top right is the left lateral view of the skin model and bottom right is the dorsal view of the skin model.

In this study, quantification of electrode localization error was also planned in the Brachycephalic and Dolichocephalic cohorts. Yet, when attempting to register the 3-D models to the brain atlas it was noted that the transformation to the CT images did not adequately match the anatomy, meaning no common reference coordinate system could be established for these head morphologies. This meant the electrode localization error could not be calculated, suggesting different brain atlases are required for brachycephalic and dolichocephalic breeds. This limitation agrees with Johnson et al. (12) as they noted that significantly more warping was required for brachycephalic and dolichocephalic breeds than mesocephalic. This presents an issue as there is no quantitative way of determining what skull conformation a dog breed has. Additionally, variation within breeds must be considered. On top of this, there are many dogs that have mixed breed heritage and may not fit into what would typically characterized as brachycephalic, mesocephalic or dolichocephalic conformations. This presents a major issue when attempting to formulate an EEG array. While the Pellegrino and Sica (8) study proposed modified EEG arrays for brachycephalic breeds and mesocephalic breeds, refinements must take into account dogs that fall into a grey area between skull conformations, as well as how to define the different categories of morphology. This means that regardless of the accuracy and repeatability of the electrode placement, it is unclear when the brachycephalic array should be used rather than the mesocephalic array.

The qualitative portion of this study revealed differences in the coverage of the EEG electrode array between dogs with different skull conformations. It was noted that brachycephalics had no coverage of the olfactory bulb with all F electrodes covering the frontal lobe. This was suspected to be due to the relatively ventral location of the olfactory bulb in this group, meaning dorsally placed electrodes could not achieve adequate coverage of this region. Conversely, all 5 of the F electrodes landed over the olfactory bulb in dolichocephalic dogs. It should be also noted that the olfactory bulb is a silent region of the brain, so neurological examination would not help to corroborate if this is where seizures are originating from. In mesocephalic breeds sometimes the Fz electrode was over the most rostral aspect of the frontal gyrus. In brachycephalic dogs, all C electrodes landed on the caudal aspect of the parietal lobe or cranial aspect of the occipital lobe. In contrast all C electrodes placed on dogs with mesocephalic or dolichocephalic skull conformations were closest to the frontal lobe. In all dogs the T electrodes were consistently over the temporal lobes. Finally, O1, O2 and Pz all covered the occipital lobe. This again suggests that the rostral electrodes in EEG electrode arrays should be modified based on skull conformation, which again raises the issue of a practical definition for skull conformation without the need for imaging. In addition to conformation, patient size should also be considered. It has previously been noted that electrode placement can be more challenging in smaller patients and may affect the accuracy of placement (11). This suggests placement of fewer electrodes may be beneficial in smaller patients, necessitating further understanding of the most important electrodes to place in such cases. With all of this considered, proposal of new EEG electrode arrays to achieve adequate cortical coverage in all dogs will likely be a complex, morphology dependent task.

Taken together, further research is required to minimize the extent of inter-observer variability. The variation in electrode placement could occur for several reasons, ranging from a misunderstanding of, or unclear, instructions for electrode placement to greater variability in dog head and brain morphology than encountered in the original dissections. One way to control for inter-placer variability would be through development of an EEG cap, like that used by Lyon et al. (18). The Lyon study demonstrated the feasibility of using elastic straps to hold EEG surface electrodes in place. Of note, they were able to place these EEGs in all animals without the need for sedation. Whilst that study is a promising step in the development of a standardized EEG cap, further investigation is required to determine the localization error of electrode placement with this method. The variability of electrode placement based on skull conformation noted in the qualitative part of this study suggests that different EEG caps will be required for different skull conformations. As well as the benefit of helping to control for inter-placer variability, EEG caps would also facilitate easier and faster electrode placement. Aside from helping to improve accuracy of localization, use of dry surface electrodes in the caps would also result in less patient discomfort compared to subdermal needle or wire electrodes. Live dog localization error could be confirmed by placing the electrodes and performing CT of the head. Whilst EEG caps may be an ideal development in the future it should be noted that even in humans there has been some variation in electrode positioning when using a fixed cap (19, 20). Attempts are currently being made to improve on the accuracy of electrode placement in human papers, with improved electrode accuracy having been demonstrated when using an augmented reality electrode guidance system compared to when using the standard 10–20 system (21).

Limitations of this study explicitly include the lack of quantification of electrode placement error in brachycephalic and dolichocephalic breeds. To do this, development of a stereotactic brain atlas with a common reference coordinate system for these specific skull conformations would be required. It is also noted that incorporation of the atlas into the brain models had to be done manually, meaning the coordinate system could be influenced by human error during registration in BrainSight. It also meant that adequate sizing of the brain atlas overlay was subjective. Additionally, in the mesocephalic cohort where EEG electrode placement error was quantified it should be noted that there was a significant age range (11-years). This is of importance as cortical atrophy is a well documented age-related change in humans, occurring in both normal individuals and those with neurodegenerative diseases (22–28). In theory cortical atrophy should impact EEG electrode placement relative to cortical landmarks due to brain shrinkage and increased CSF: brain volume (27). In order to determine the effect of the impact of this, further studies involving a larger number of mesocephalic dogs should be performed. This would facilitate determination of the effect of age-related changes on EEG cortical coverage and electrode placement error. Finally it is of note that there is significant diversity within each cohort despite controlling for skull conformation. Given the small sample sizes, this may have had an impact on the results of this study. Larger follow up studies would enable further investigation into the impact of these variables on electrode placement.

The under-use of EEG in veterinary medicine likely has significant clinical implications. Seizures are likely under-diagnosed as there is, at best, only moderate agreement between veterinary observers as to the nature of a paroxysmal event (29). Seizures are also likely under-reported, as it was demonstrated that there is only weak agreement between frequency of seizures reported by caregivers and ictal paroxysmal discharges on EEG (30). The incorporation of EEG findings into the definitions of canine epilepsy syndromes will strengthen comparative investigations and improve treatment specificity for dogs. This is important because some epilepsy syndromes in people are worsened by specific anti-seizure medications. Such findings have so far not been described in veterinary patients. Further understanding of epilepsy syndromes may be significant as it is currently noted that 20–30% of canine epilepsy patients are considered refractory to medications and are poorly controlled on anti-seizure medications (7, 31). It is possible that a greater understanding of these epilepsy syndromes may help to tailor treatment and improve efficacy when treating dogs with idiopathic epilepsy. The consensus may be shifting, though: a 2024 American College of Veterinary Internal Medicine (ACVIM) consensus statement recommended that EEG be performed to identify patients experiencing non-convulsive status epilepticus (32). This builds on previous recognition that a major EEG strength is to identify non-convulsive status epilepticus in veterinary patients (33–35).

The lack of a standardized EEG array in veterinary patients is a major gap in our knowledge. It limits the ability to perform accurate source localization in dogs and cats, to identify veterinary epilepsy syndromes, and would facilitate the development of epilepsy surgery. Given the variability of electrode location due to skull conformation, the lack of definitive measurements to determine skull conformation and the F7 and F8 electrode placement issue, this study raises the question of whether it will ultimately be possible to formulate a standardized EEG array. In people, standardized EEG arrays have been established with known electrode placement error, however in dogs there is much more variability in skull shape than there is in people. This makes the development of a standardized EEG array in dogs particularly challenging. Whilst expensive and somewhat impractical, it is possible that the use of CT or MRI to confirm relative electrode placement will be required in all canine patients undergoing EEG for the purposes of accurate source localization.

In conclusion, this was the first study that set out to validate the EEG electrode array proposed by James et al. (11) and the first to attempt the quantification of EEG electrode placement error in dogs. Imaging based neuronavigation made this possible through the establishment of a common reference coordinate system. As these virtual 3-D models were instrumented by a single observer, future studies in live dogs would be required to establish average inter-observer variance. This study highlights the current knowledge gaps and potential challenges that may be faced in the future while aiming to develop a standardized EEG array in dogs due to the differences in brain morphology in brachycephalic, mesocephalic and dolichocephalic breeds. Given the differences in coverage between groups, different EEG electrode arrays will likely be required based on skull conformation. Currently, there is no quantitative way of defining a dog’s skull conformation, therefore future study is required to achieve this, which, in turn, would facilitate morphology-specific array selection to solve the inverse problem in the canine brain.

## Data Availability

The original contributions presented in the study are included in the article/Supplementary material, further inquiries can be directed to the corresponding author.
