# Supplementary material for: Scalp electrode placement accuracy for the canine electroencephalography array
Source: Front Vet Sci. 2025 Jun 20;12:1543836. doi: 10.3389/fvets.2025.1543836 (PMC12226243; doi:10.3389/fvets.2025.1543836)
Supplement: Supplementary file 1 [file Table_1.DOCX]

Supplementary Material

# Supplementary Data

MATLAB code scripts used to plot the PCA overlays and create the 3D brain model to place them on.

Calculate PCAs:

% Read in spreadsheet

filename=uigetfile('*.xlsx','Load File of Sensor Locations');

data=table2array(readtable(filename));

% Number of sample coordinates is # of rows minus 1 for mean in last row

n=size(data,1)-1;

% Number of sensors (electrodes) is # of columns / 3

n_sensors=size(data,2)/3;

% Mean is last row

data_mean=data(n+1,:);

data=data(1:n,:);

% Pre-allocate variables for direction vectors & standard deviations

PCA_directions=zeros(3,n_sensors*3);

PCA_stdevs=zeros(1,n_sensors*3);

% For each sensor, perform PCA on the 3 co-ordinates

for i=1:n_sensors

Xindx=(i*3-2); Zindx=i*3;

[dirs,score,vars]=pca(data(:,Xindx:Zindx));

PCA_directions(:,Xindx:Zindx)=dirs;

PCA_stdevs(:,Xindx:Zindx)=sqrt(vars);

end

% Write out spreadsheet of PC analysis

output_spreadsheet=array2table([PCA_directions; PCA_stdevs]);

filename=uiputfile('*.xlsx','Save File of PC Analysis');

writetable(output_spreadsheet,filename)

Visualise PCAs:

function visPCAs(means,directions,stdevs)

n_sensors=size(stdevs,2)/3;

figure(1)

hold on

axis equal

grid on

xlabel('X'); ylabel('Y'); zlabel('Z')

colours='rgbcmyk';

for i=1:n_sensors

% x 1st col, y 2nd col, z 3rd col

x_indx=i*3-2;

y_indx=i*3-1;

z_indx=i*3;

% Mean co-ordinate of sensor

x_mean=means(x_indx);

y_mean=means(y_indx);

z_mean=means(z_indx);

% Plot axes for only first 2 largest components (3rd is redundant)

for PC=1:2

% Each column is a PC direction vector

% 3D direction of PC: x 1st row, y 2nd row, z 3rd row

x_dir=directions(1,i*3+PC-3);

y_dir=directions(2,i*3+PC-3);

z_dir=directions(3,i*3+PC-3);

% Length (one standard deviation) of component

r=stdevs(i*3+PC-3);

% Plot +/- axis of component, scaled by length of standard deviation

plot3(r*[-x_dir x_dir]+x_mean, r*[-y_dir y_dir]+y_mean, r*[-z_dir z_dir]+z_mean, 'k');

end

% Ellipsoid of unit standard deviation contour

[pcasurfX, pcasurfY, pcasurfZ]=ellipsoid(0, 0, 0, stdevs(x_indx), stdevs(y_indx), stdevs(z_indx));

sz_mesh=size(pcasurfX);

sz_mesh2=sz_mesh(1)*sz_mesh(2);

% Rotate ellipsoid by directions of components

rot=directions(:,i*3+(-2:0))';

pcasurfXYZ=[reshape(pcasurfX,[sz_mesh2,1]) reshape(pcasurfY,[sz_mesh2,1]) reshape(pcasurfZ,[sz_mesh2,1]) ];

pcasurfXYZ_t=pcasurfXYZ*rot;

pcasurfX_t=reshape(pcasurfXYZ_t(:,1),sz_mesh)+x_mean;

pcasurfY_t=reshape(pcasurfXYZ_t(:,2),sz_mesh)+y_mean;

pcasurfZ_t=reshape(pcasurfXYZ_t(:,3),sz_mesh)+z_mean;

% Finally plot (render) the ellipsoid

%mesh(pcasurfX_t, pcasurfY_t, pcasurfZ_t,'FaceAlpha','0.1','EdgeColor',colours(mod(i-1,7)+1));

%surf(pcasurfX_t, pcasurfY_t, pcasurfZ_t,'FaceAlpha','0.1','EdgeColor',colours(mod(i-1,7)+1),'FaceColor',colours(mod(i-1,7)+1));

surf(pcasurfX_t, pcasurfY_t, pcasurfZ_t,'FaceAlpha','0.2','EdgeColor','none','FaceColor',colours(mod(i-1,7)+1));

end

Visualise brain:

filename=uigetfile('*.stl','Load 3D CAD File');

TR=stlread(filename);

% Create surface

hsurf=trisurf(TR,'FaceColor',[0.8 0.8 0.8],'EdgeColor','none','FaceAlpha',0.7);

% Light up suface - one in front, one in back

l1=light('Position',[0 80 80],'Color',[0.9 0.9 0.9]);

l2=light('Position',[0 -200 160],'Color',[0.7 0.7 0.7]);

Plot Locs:

function plotLocs(locs)

n_sensors=size(locs,2)/3;

figure(1)

hold on

axis equal

grid on

xlabel('X'); ylabel('Y'); zlabel('Z')

colours='rgbcmyk';

for i=1:n_sensors

% x 1st col, y 2nd col, z 3rd col

x_indx=i*3-2;

y_indx=i*3-1;

z_indx=i*3;

plot3(locs(:,x_indx),locs(:,y_indx),locs(:,z_indx),strcat('.',colours(mod(i-1,7)+1)),'MarkerSize',5);

end
